# Cancer surgery induces inflammation, immunosuppression and neo-angiogenesis, but is it influenced by analgesics?

**DOI:** 10.12688/f1000research.2-102.v1

**Published:** 2013-04-03

**Authors:** Patrice Forget, Olivier Simonet, Marc De Kock

**Affiliations:** 1 Departments of Anesthesiology, Cliniques Universitaires Saint-Luc, Université Catholique de Louvain, Brussels, Belgium; 2 Department of Anesthesiology, Centre Hospitalier Wallonie-Picarde, Tournai, B-7500, Belgium

## Abstract

Surgery remains a main part of the treatment of most solid tumors. Paradoxically, rapid disease progression may be a consequence of surgery in patients presenting with a dysregulated inflammatory response, and increased angiogenesis consequent to a suppressed antitumoral immune response. Physicians taking care of cancer patients should be aware of the important findings that indicate that analgesic techniques could play a role in these phenomena.

## Introduction

The natural history of cancer is a complex and rapidly evolving field. For example, in breast cancer, the growth of the primary tumor and the dissemination of neoplastic cells are linked, at least in part, to inflammation leading to immune dysfunction and an/or increased angiogenesis ^1, 2^. For breast cancer, as well as for most solid tumors, surgery remains a main part of the treatment. However, paradoxically, the surgical period, and the associated inflammatory reaction, is itself a high risk factor for the development of metastases and this phenomenon may be explained by the rapid release of inducers of angiogenesis concomitant to a profound immunosuppression ^1, 2^. One example, sometimes observed, is the rapid postoperative development of additional tumors and metastasis when a primary tumor is surgically removed ^2^.

Other factors can accentuate this phenomenon, including the metabolic and hormonal changes that occur and are determined by the inflammatory/catecholaminergic "stress reaction" to surgery ^3^. To counteract these effects, perioperative physicians, including anesthesiologists, surgeons and oncologists, must help the patient to maintain homeostasis against the consequences of both cancer and tissular attrition. Anesthetic and analgesic techniques are one part of this strategy, but their effects, however important, are different and not well understood. Indeed, these drugs may influence immunity and tumor development, either directly by interfering with cellular mechanisms (e.g. cell apoptosis) or indirectly by interactions with the endocrine and sympathetic systems.

In this paper, we discuss the consequences of perioperative inflammation in cancer surgery on immunity and angiogenesis. Secondly, we describe why analgesic techniques may play a role in these phenomena.

## Perioperative inflammation-related immunosuppression and neoangiogenesis are seen in cancer patients at risk of relapse

The early existence of dormant metastasis is matter of debate ^2^. One argument is the kinetics observed in cancer recurrence after breast cancer surgery. Indeed, when analyzing the timing of the relapse of patients under endocrine therapy, recurrences occur gradually over the first 10 to 15 years. In contrast, in women not treated with endocrine therapy (i.e. with estrogen-receptor-negative tumors), the majority of cancer relapses occur in the first two years ^4^. This suggests that these tumor cells have been maintained in a "dormant" state in the first group of women, whereas they may be present early in both groups ^5^.

The risk for these patients in undergoing surgery is that rapid growth of these cells could be induced through perioperative inflammation. Indeed, following a surgical trauma, a great, but short-lasting, inflammation is correlated with a potent immune response that precedes a longer duration of immunosuppression ^1^. The role of this immunosuppression is probably to minimize the intensity of the proinflammatory response, and reduce the risks of autoimmune disorders and/or necrosis of tissue ^6, 7^. Locally and throughout the body, the release of cytokines underlies the initiation, maintenance and regulation of the inflammatory response. After tissue injury, monocytes and macrophages rapidly release interleukin 1 (IL-1) and tumor necrosis factor alpha (TNF-α). IL-1 stimulates and maintains the secretion of other cytokines such as interleukin 6 (IL-6) that are major mediators of the systemic effects of the stress response. The production of adrenocorticotropic hormone (ACTH) and cortisol are stimulated and are regulated by a negative feedback system ^8^.

The tissue damage caused by surgery is also likely to generate pain. This causes the secretion of endogenous opioids that provide analgesia of short duration by their peripheral and central effects ^9^. Peripherally, the presence of opioid receptors on immune cells allows β-endorphins to have a direct effect on the proliferation, migration and cytotoxicity of these cells ^9^. Some neurotransmitters, including substance P, can interact with the pain pathways. At the central level, active pain regulatory mechanisms act via the periaqueductal gray matter of the midbrain and through the β-endorphin and catecholaminergic pathways. Norepinephrine inhibits natural killer (NK) cell activity via the β-2 receptor. The cholinergic system and the vagus nerve play a role that is often opposite to that of the sympathetic system ^10^.

In turn, the brain monitors and controls these loops of inflammation. The hypothalamic-pituitary, cortex and cerebellum are involved in the control of lymphoid organs, especially the spleen and many adrenergic receptors are present on B and T lymphocytes, macrophages, neutrophils and NK cells. Finally, the endocrine system interacts with the brain, immune cells and most organs ^6, 7^. Glucocorticoids, for example, have anti-inflammatory properties but are essential for normal immune response. Prostaglandins and prostacyclin, particularly PGE2, are produced, among others, by dendritic cells and macrophages. They have a major role in the regulation of immunity in general and NK activity in particular. In addition, PGE2 plays a major role in the stimulation of epithelial cell proliferation, inhibition of apoptosis, production of mutagens and stimulation of angiogenesis ^11^. This may explain the high incidence of overexpression of COX-2 in breast cancer, as in other cancers, and higher aggressiveness of these tumors ^11^.

Neo-angiogenesis is probably a major step in the induction of growth of a dormant metastasis. Indeed, it appears that solid tumors cannot grow larger than 2–3 mm in diameter unless they induce their own blood supply ^12^. The expression of the angiogenic phenotype, physiologically to promote wound healing, is a complex process that depends on a number of cellular and molecular events, including degradation of the surrounding basement membrane, migration of endothelial cells, cell proliferation, the formation of tube-like structures and the maturation of these endothelial-lined tubes into new blood vessels ^13^.

As a consequence, most of the modifications in the immune system and the angiogenesis phenotype are linked to the degree of tissue injury and its consequent inflammation. The logical strategy is then to promote minimally invasive surgery that is designed to limit these impacts, to maintain homeostasis ^14^ and reduce the stress response ^8^. These arguments have led some authors to consider that minimally invasive procedures might be favorable in terms of immunity when compared with invasive procedures due to the need to manage other changes driven by the "secondary aggressions", i.e., hypothermia, hemorrhage, and psychological factors such as anxiety ^1^.

## Effect of analgesics on inflammation, anticancer immunity and angiogenesis

A possible way to influence the perioperative inflammatory reaction and their consequences for immune cells and angiogenesis is by modifying analgesic techniques. This could have an important impact on patient outcome following surgery.

Macrophages, T lymphocytes, NK T and other cells and many cytokines are involved in the defense mechanisms of nonspecific immunity. Of these, the role of NK cells in defense against infection and the development of tumor cells ^1, 15– 17^ has been largely demonstrated. The study of animal models has helped us to understand their role in the mechanisms behind perioperative anti-metastatic protection, but also their vulnerability ^16– 18^. In humans, a significant correlation between NK activity and patient prognosis is related to the development of metastases ^19^. The strong depression of the cytotoxicity of the NK cells in the perioperative period may significantly alter the defense mechanisms of patients ^1^.

Analgesic techniques that reduce the inflammatory response may be favorable in terms of immunity ^8^. Recent data suggest that the analgesic techniques (intravenous opioids, non-steroidal anti-inflammatory drugs (NSAIDs) and locoregional analgesia) could have an impact on the long-term prognosis after cancer surgery ^20, 21^, including for breast cancer ^22, 23^. Opioids are the drugs most studied in the context of the perioperative immune response. In the absence of pain, morphine induces a decrease in NK activity ^25^. It is not clear whether it is the opioids, but possibly also the withdrawal of opioid therapy, that is responsible for opioid-induced immunosuppression ^24^. Nevertheless, pain itself induces a significant immune response. Because this response leads to a significant degree of immunosuppression, postoperative analgesia is, by itself, immunoprotective ^1^. Morphine therefore allows the maintenance of NK activity and protection against metastasis in animal pain models ^25^. In humans, similar arguments can be used to offer the lowest effective dose of opioid ^24^. Data on synthetic opioids have revealed a similar phenomenon with fentanyl and sufentanil, probably mediated via the µ-opioid receptor. Nevertheless, differences linked to selective µ1-activation by synthetic opioids, in contrast to µ3-activation by morphine, could explain the more favorable profile of morphine and may merit further investigation ^25^. Opioids could also affect angiogenesis and the growth of tumors. In the perioperative period and after tissue injury, important angiogenic signals, including epithelial growth factors (VEGF), act via receptor tyrosine kinases and G-protein-coupled receptor (GPCR). This may have a major importance as morphine can transactivate these GPCR, increasing neo-angiogenesis ^13^.

Prostaglandins are another major perioperative influence on immunity and angiogenesis and the promotion of tumor growth ^26^. The data concerning the maintenance of NK activity by NSAIDs are encouraging, in some, but only a few human clinical studies. In a retrospective study, we showed that receiving ketorolac, a NSAID, just before surgery, was an independent factor associated with longer recurrence-free survival after breast cancer surgery ^22^. These results may be explained by the fact that prostaglandin E2 (PGE2), released by monocytes and dendritic cells in order to regulate the inflammatory cascade, profoundly depresses cellular antitumoral immunity, i.e. NK activity ^27^. This suppression of NK activity, and possibly an initial flare-up of angiogenesis, dissipates quickly after the removal of the prostaglandins. There may then be a short therapeutic window when NSAIDs may have a potent impact on the oncological outcome ^22^.

It seems clear that regional anesthesia, particularly central blocks, are associated with anti-inflammatory effects, and allow the protection of anticancer immunity, including NK activity, after major surgery ^28^, but this effect has not been demonstrated after minor surgery ^29^. It is possible that this effect is responsible for a lower incidence of recurrence after surgery for breast, colon or prostate cancers, however this is an unresolved debate due to poor methodology and lack of perioperative immune monitoring ^20, 21, 23, 30– 33^.

Anesthesiologists are using many other drugs that are not necessarily associated with analgesic effects. Data are sparse and mostly inconclusive concerning barbiturates, halogenated gases, propofol and etomidate. It seems likely that ketamine, a widely used co-analgesic, has a dose-dependent effect, being protective at low doses during a painful stimulus and potentially harmful at high doses in the absence of surgery ^24^. One interesting topic is the potential protective role of alpha-2-agonists, such as clonidine, typically used as co-analgesic during surgery on perioperative NK activity ^24^.

## Is it time to change our pain management practices?

A goal of optimal control of pain may appear to be obvious, but it still remains a major concern for perioperative physicians. The quality of analgesia is facilitated by combinations of molecules (NSAIDs and anti-hyperalgesics such as ketamine) and techniques (intravenous, locoregional with local anesthetics). At this time, it is not clear whether these techniques may improve oncological outcome alone, indirectly by the avoidance of opioids, or both. Nevertheless, they improve pain management, postoperative rehabilitation and the prognosis for patients. These reasons remain the main argument to recommend such analgesic approaches before definitive conclusions on their influence on the oncological outcome ^8, 14^.

Moreover, it appears that high doses of opioids are carriers of long-term side effects that are probably underestimated (including opioid-induced hyperalgesia). The study of the role opioids play in the increase in angiogenesis and postoperative immunosuppression is of particular importance in oncological surgery, knowing that NK activity could be a prognostic criterion ^13, 19^. Taking into account that they improve pain management, it is appropriate to propose opioid-sparing strategies, such as locoregional techniques and sympathetic modulation using intravenous α-2 agonists ^24^.

## Conclusions

Understanding cancer development mechanisms is a major goal of clinical research and may lead to the development of strategies to counter the multiple factors involved in cancer pathology. It is very important to study all aspects of the problem, permitting the development of strategies associated with a higher long term survival after cancer surgery. The data available already affirm that the immune system and the angiogenesis phenotype play key roles in anticancer defenses, that the potential influence of surgery-related inflammation is large and that the effects of drugs and the techniques of analgesia may be important. Perioperative physicians must be aware that an optimal analgesic strategy may have a long-term impact on patient outcome.
