# Supplementary material for: Cancer surgery induces inflammation, immunosuppression and neo-angiogenesis, but is it influenced by analgesics?
Source: F1000Res. 2013 Apr 3;2:102. [Version 1] doi: 10.12688/f1000research.2-102.v1 (PMC3752648; doi:10.12688/f1000research.2-102.v1)
Supplement: Peer review history

# Peer review: Referee response for version 1

**Published:** 2013-05-09

**Authors:** Dan Benhamou

**Affiliations:** Bicetre Hospital, Paris, France

To the Editor,

First, note that it is extremely difficult to write detailed comments as lines are not numbered. Please modify the text to facilitate reviewers’ work.

This is a nice review of the role of analgesia on postoperative outcome after cancer surgery. As stated by the authors, we still need confirmatory data and the present status of our knowledge only suggests a speculative effect, even if most studies concur to suggest a beneficial effect of a well-performed postoperative analgesia. It would also be important to note that all additional factors of a well-performed anesthesia (i.e. avoidance of hypotension or of hypothermia, treatment of anemia, prevention of hyperglycemia…) may also be factors that could improve postoperative outcome after cancer surgery.

That said, the reviewer has several minor comments to add:

Page 2, 2nd column, L12 of 1st paragraph: is instead of are (the subject of the sentence is “The production of…”)

Page 2, 2nd column, L2 of 3rd paragraph: hypothalamic-hypopituitary complex/system/unit/axis… (choose one word)

Page 3, 1st column, L9 of 2nd paragraph: add “reduced”? (…with invasive procedures due to the reduced need…)

Page 3, 1st column, L1 of 5th paragraph: in this sentence and all along the text, use “regional” instead of “locoregional”

Page 3, 1st column, L10 and following lines of 5th paragraph: paragraph unclear: reorganize sentences to present first the beneficial effects of opioids (reduced pain) and then their harmful effects to facilitate reading

Page 3, 2nd column, L3 of 3rd paragraph: what do the authors mean by “protection of anticancer immunity”?

Page 4, 1st column, L10 of 2nd paragraph: it appears somewhat surprising that alpha-2 agonists are promoted with some apparent strength given the fact that our knowledge on these drugs in this context are not better that for other agents. Please modify the sentence to attenuate the strength of this recommendation.

I have read this submission. I believe that I have an appropriate level of expertise to confirm that it is of an acceptable scientific standard.

# Peer review: Referee response for version 1

**Published:** 2013-05-07

**Authors:** Erxi Wu

**Affiliations:** North Dakota State University, Fargo, ND, USA

This is an interesting research topic. It describes that cancer surgery induces inflammation, immunosuppression, and angiogenesis. Also these phenomena can be influenced by analgesics. As we know, Galen (Aelius Galenus) removed some tumours surgically, but he generally believed that cancer was best left untreated. The underlying mechanisms were not clear then. The research community can benefit from this well-written paper as this paper has updated the information for this topic.

This paper can be improved if the mechanisms would have been discussed in depth. After cancer surgery, HIF-1alpha and MMPs may also be up-regulated. This paper has not mentioned them. Thus, it would be helpful if authors search and study more literature. 

I have simply searched some literature, I think the following papers should be cited:

Tavare AN *et al*. Cancer recurrence after surgery: direct and indirect effects of anesthetic agents. *Int J Cancer*. 2012 doi: 10.1002/ijc.26448

Retsky M *et al*. NSAID analgesic ketorolac used perioperatively may suppress early breast cancer relapse: particular relevance to triple negative subgroup. *Breast Cancer Res Treat*. 2012 doi: 10.1007/s10549-012-2094-5

Deegan CA *et al*. Anesthetic technique and the cytokine and matrix metalloproteinase response to primary breast cancer surgery. *Reg Anesth Pain Med*. 2010 doi: 10.1097/AAP.0b013e3181ef4d05

Thaker PH *et al*. Chronic stress promotes tumor growth and angiogenesis in a mouse model of ovarian carcinoma. *Nat Med*. 2006 doi: 10.1038/nm1447

I have read this submission. I believe that I have an appropriate level of expertise to confirm that it is of an acceptable scientific standard.

# Peer review: Referee response for version 1

**Published:** 2013-04-08

**Authors:** Alain Borgeat

**Affiliations:** Orthopedic University Hospital Balgrist, Zurich, Switzerland

There are more and more clues that inflammation, immune system and cancer recurrence may be directly associated. Therefore, this manuscript is very actual. It is well constructed and raises basic questions concerning the expected future adaptations of the management of the perioperative period.

I have read this submission. I believe that I have an appropriate level of expertise to confirm that it is of an acceptable scientific standard.
